# Censoring, Competing Events, and Multistate Models: Comment on Beyersmann et al. “Hazards Constitute Key Quantities for Analyzing, Interpreting and Understanding Time‐to‐Event Data”

**DOI:** 10.1002/bimj.70153

**Published:** 2026-07-14

**Authors:** Malka Gorfine, Daniel Nevo

**Affiliations:** ^1^ Department of Statistics and Operations Research Tel Aviv University Tel Aviv Israel

**Keywords:** causal inference, censoring, clinical trials, multistate models, transportability

## Abstract

Beyersmann et al. propose a functional interpretation of hazards, viewing them as evolving quantities describing the entire event process rather than as pointwise causal contrasts. In this commentary, we elaborate on the implications of this view for causal inference in modern clinical trials with survival outcomes. We emphasize how censoring, competing events, and multistate structures shape not only identifiability but also the definition and transportability of hazard‐based estimands. We highlight that, even within a functional framework, censoring mechanisms may implicitly determine the statistical estimand through time‐dependent weighting, with direct implications for generalizability across studies and populations. We further discuss how these issues are amplified in competing‐risks and multistate settings, where causal interpretation requires careful consideration of intercurrent events and selection induced by post‐randomization state occupancy.

## 1 Introduction

We congratulate Beyersmann et al. (2025) on their thought‐provoking paper, which offers a timely contribution to the ongoing discussion about the role and interpretation of hazard functions in event‐history analysis within a causal framework. Beyersmann et al. (2025) use the term functional in a process‐oriented sense: hazards are interpreted as full time‐dependent functions describing the evolution of the event process under each intervention. Contrasts between certain functionals of the entire hazard function (e.g., a survival probability), rather than pointwise hazard ratios, capture the causal effect as a dynamic feature of the underlying stochastic process. By adopting a functional and process‐based perspective, the authors illuminate both the strengths and the inherent limitations of using hazards to characterize causal effects in time‐to‐event analyses. Their work clarifies how hazard‐based quantities can be meaningfully interpreted, especially when viewed as components of a counting‐process representation of the data‐generating mechanism.

While the hazard function remains a central and indispensable tool for analyzing censored survival data, it also presents interpretational difficulties from a causal standpoint. In particular, the instantaneous or “local” hazard ratio is challenging to interpret causally, as it compares the instantaneous event rate between two subpopulations that are likely to differ. This raises an important conceptual question: How can valid causal inference about survival probabilities be achieved when the modeling framework is built upon such non‐causal local quantities? The counting‐process representation presented in Figure 1B of Beyersmann et al. (2025) illustrates that causal comparisons of survival functions can still emerge from hazard‐based formulations when the outcome node is the counting process.

**FIGURE 1:**
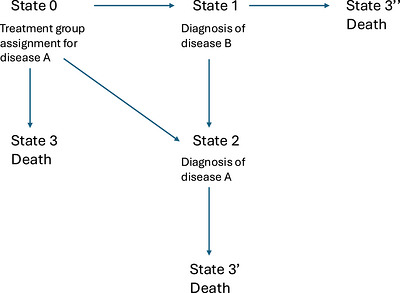
Constructed example of a multistate disease‐progression process in a randomized clinical trial of a treatment for disease A. From the initial disease‐free state 0, participants may develop disease A (State 2) or disease B (State 1), progress from disease B to disease A (1→2), or die before or after diagnosis. Intercurrent (disease B) and competing events (death: State 3 or State $3^{\prime \prime }$) illustrate the complexities of defining and estimating causal treatment effects on the time to disease A.

Our discussion highlights selected complementarities between the arguments presented by Beyersmann et al. (2025) and causal reasoning in survival analysis. In particular, we focus on three issues that are central for the interpretation of hazards in modern clinical‐trial time‐to‐event analyses: censoring, competing events, and multistate structure. We believe that the paper of Beyersmann et al. (2025), together with the ensuing discussion, will stimulate further research on principled causal inference for time‐to‐event outcomes, especially under competing risks or multistate setting in general.

## 2 What, If Yes Censoring?

Beyersmann et al. (2025) emphasize that the notion of censoring does not necessarily lead to a hypothetical “world without censoring.” The argument is based on the notion that, under conditional independent right censoring, given X, and including administrative censoring, hazard functions are identifiable from censored data and are the building blocks of survival distributions. We would like to emphasize that, even in this familiar independent‐right‐censoring setting, censoring can affect the statistical target of model‐based summaries and therefore the interpretation and comparability of study results.

We first highlight an implication of censoring when parametric or semiparametric models are employed. Let $h_{0}\left(t\right)$ and $h_{1}\left(t\right)$ be the true hazard functions in the untreated and the treated groups, respectively, and write

$$h_{1}\left(t\right)=h_{0}\left(t\right)\exp\left\{\beta \left(t\right)X\right\}\;,$$

where X denotes the binary treatment arm indicator, with 0 and 1 representing the two treatment groups. Suppose that the analyst fits the misspecified working model

$${\tilde{h}}_{1}\left(t\right)={\tilde{h}}_{0}\left(t\right)\exp\left(\gamma X\right)\;.$$

Here, misspecification refers to fitting a working proportional hazards model with a constant log‐hazard ratio γ, although the true hazards ratio is time‐varying, β(t). Under this misspecification, the Cox model estimator $\hat{\gamma }$ converges to a weighted average of the time‐varying contrast β(t) over t (Anderson and Fleming 1995; Lagakos and Schoenfeld 1984). These weights are determined by the evolving event and censoring processes through the at‐risk sets; therefore, censoring changes both the size and composition of the risk sets over time and, consequently, the implicit emphasis placed on different values of β(t). This has direct implications for transportability. Consider two similar studies conducted in different populations that share the same underlying control hazard function and the same time‐varying coefficient β(t), but differ in their censoring mechanisms. The induced weights over time will differ, yielding distinct effective parameters γ and, consequently, distinct limiting values for the estimators $\hat{\gamma }$. As a result, the parameter estimated in one population cannot be transported to another without accounting for differences in the censoring, even in this ideal setting with identical baseline hazards. Even in the same population, trials with different follow‐up windows or administrative censoring times may target different model‐based summaries, because different portions of the time‐varying treatment contrast receive different weights. This observation is especially pertinent because violations of the proportional hazards assumption are common in medical studies (Bardo et al. 2024); hence, caution is warranted when interpreting or generalizing simplified hazard‐based summaries across populations. For the causal interpretation of proportional hazards summaries, see also Stensrud and Hernán (2020). In general, the censoring distribution determines how information about the time‐varying hazard ratios β(t) is weighted over time, thereby defining the estimand itself. Consequently, under the nonproportional hazards, the estimated coefficient $\hat{\gamma }$ should not be interpreted as a universal treatment parameter. Transporting or comparing this coefficient across populations or trials requires the follow‐up window, administrative censoring time, event process, and additional censoring mechanisms either to be aligned or to be explicitly accounted for in the target estimand and analysis. This conceptual problem could be avoided, for this particular summary, by defining a common analysis horizon and considering only administrative censoring at that prespecified follow‐up time, with no additional censoring mechanisms. If we define the causal estimand as the risk difference at the end of the trial and fit the misspecified model, then the resulting estimators $\hat{\gamma }$ from the two trials will induce the same limiting estimate of the risk difference. Consequently, both trials asymptotically agree on the target risk‐difference estimand. Although this common limit may be biased due to model misspecification, the two otherwise identical trials will asymptotically agree in their results.

Our discussion complements the functional perspective advocated by Beyersmann et al. (2025) by emphasizing that, even within this functional framework, the weights implicitly induced by censoring determine the statistical estimands and their interpretation. Thus, while the functional formulation remains valid for describing within‐population dynamics, generalizability to the underlying study population and transportability across populations require careful attention to the censoring mechanisms, as they may fundamentally shape the estimands.

To recap this section, recognizing censoring as part of the design and definition of the estimand, rather than a nuisance feature, clarifies that hazard‐based parameters represent context‐dependent summaries of causal effects, whose magnitudes depend on the follow‐up and censoring scheme of the study rather than on a fixed, universal biological constant. In particular, the estimated log‐hazard ratio $\hat{\gamma }$ reflects not only biological dynamics but also design‐ and subgroup‐specific censoring patterns (Schumacher et al. 1987; Stensrud and Hernán 2020). This is a crucial source of non‐transportability that links directly to broader concerns about reproducibility in modern survival analysis.

## 3 What, If No Competing Events?

Even if censoring were absent, event probabilities, particularly in competing‐risk settings, depend on multiple cause‐specific hazard functions simultaneously. Thus, while cause‐specific hazards remain fundamental building blocks of the event process, and Nelson–Aalen cause‐specific estimators are defined by treating other events as censoring, their interpretation does not rely on imagining a censoring‐free world. Rather, it is based on understanding how the collection of cause‐specific hazards jointly determines event probabilities. In a general multistate formulation, transition probabilities are functionals of the relevant transition intensities and event history; no Markov assumption is intended unless it is stated explicitly.

Unlike censoring, competing events cannot generally be regarded as removable nuisances. While it is often meaningful to conceptualize a hypothetical world without censoring, imagining a setting in which competing events have been eliminated is frequently impossible or scientifically uninformative (Prentice et al. 1978). The controlled direct effect provides a transparent potential–outcomes formulation of such a quantity by explicitly defining, in a discrete‐time setting, the effect of an intervention under the hypothetical elimination of competing events (Young et al. 2020). Precisely because this definition requires envisioning an intervention that prevents competing events from occurring, it clarifies that the resulting estimand often lacks scientific relevance. This issue should also be central when contrasting two seemingly statistically similar phenomena: dependent censoring and competing events. In both cases, we can identify from the observed data the cause‐specific hazard of the event of interest, but in the former case we are typically interested in estimands defined had there been no censoring, and in the latter such an estimand is unlikely to be relevant to the scientific inquiry, and other estimands are typically considered.

The difference between the cumulative incidence functions of the event of interest under the two treatment strategies, termed the total effect by Young et al. (2020), compares populations defined at baseline. This cumulative‐incidence contrast captures all pathways by which treatment affects the probability of the event of interest, including pathways operating through the competing event. It may be different than zero if the treatment modifies the cause‐specific hazard function of the competing event, without changing the cause‐specific hazard function of the event of interest. The separable‐effects framework, closely related in spirit to mediation analysis, provides a way to define component‐specific treatment effects in the presence of competing risks. It does so by conceptualizing the treatment as decomposable into distinct components, one influencing the event of interest and another influencing the competing event (Martinussen and Stensrud 2023; Robins and Richardson 2010; Stensrud et al. 2022). This framework is most compelling when the treatment can be decomposed into scientifically meaningful components, ideally corresponding to distinct mechanisms or biological pathways. When such components are conceptually meaningful but not directly implementable, separable effects may still be useful as estimand‐defining thought experiments; however, if no meaningful component decomposition can be specified, their scientific interpretation is limited. Building on the competing‐risks causal framework of Stensrud et al. (2022) and Young et al. (2020), formulated separable effects in discrete time and related continuous‐time formulations were subsequently developed by Martinussen and Stensrud (2023).

Similarly, the question posed by Beyersmann et al. (2025), whether one can intervene to reduce the disease‐outcome process while leaving the adverse‐event process unchanged, is meaningful as a direct intervention only when the treatment can be decomposed into scientifically interpretable components whose effects on the relevant event processes are well defined.

It is worth noting that the challenge of defining meaningful effects in the presence of competing events is not unique to time‐to‐event outcomes and arises even in simpler, censoring‐free multinomial regression settings (Nevo et al. 2021; Sasson et al. 2025). When the outcome is a categorical variable composed of mutually exclusive event types, a treatment reducing the risk of one event (say, Event 1) may effectively increase the risk of other event types (say, Event 2), even in the absence of any mechanism explaining this increase other than the reduction of the risk in Event 1.

The above discussion does not render cause‐specific hazard functions useless; quite the contrary, it underscores their key role in analyzing causal effects, as they are the fundamental quantities identifiable from censored data and are routinely estimated. For example, cumulative incidence functions are estimated by combining the cause‐specific hazard for the event of interest with the overall survival function, which is determined by all cause‐specific hazards (Aalen and Johansen 1978; Martinussen and Stensrud 2023). Consequently, in competing risks settings, limitations to transportability and generalizability arise not only from differences in censoring mechanisms but also from differences in the hazards of competing events across populations.

## 4 Multistate Settings

While Beyersmann et al. (2025) argue within a competing‐risk framework, the same conceptual issues arise, and are often amplified, in multistate models, whose use has expanded rapidly in the analysis of clinical‐trial data (Bakunina et al. 2022; Beyer et al. 2020). In many contemporary trial designs, participants may experience multiple intermediate or recurrent events before the terminal outcome of interest. These issues arise in oncology and also in non‐oncological chronic and nonfatal disease settings, where patient‐relevant trajectories such as pain, relapse, disability, hospitalization, treatment complications, or graft‐versus‐host disease may be central. For example, Le‐Rademacher et al. (2018) applied a multistate model to CALGB 10603, a randomized trial in newly diagnosed acute myeloid leukemia, with states corresponding to diagnosis at randomization, first complete remission, relapse, and death. This example illustrates how treatment effects may be studied through intermediate disease states, rather than only through a terminal endpoint. The need to capture these dynamic event sequences has led to a surge in the use of multistate models, which provide a coherent modeling framework for representing clinical trajectories through transition‐specific hazards (Gorfine et al. 2025; Therneau et al. 2024; Xia et al. 2016). However, this increased modeling flexibility also magnifies the challenges of causal interpretation.

Such models allow for a richer description of clinical pathways and are now routinely applied to evaluate composite and sequential endpoints in phase III trials, adaptive designs, and long‐term follow‐ups (Bhattacharjee et al. 2024; Manevski et al. 2022). Nevertheless, defining estimands for multistate processes that are also informative about the scientific question of interest is challenging (see, e.g., Bühler et al. 2023; Nevo and Gorfine 2022; Zehavi et al. 2025) and warrants a separate extended discussion. The challenges related to censoring and competing events persist and may be even more pronounced in multistate models. In particular, a multistate estimand must specify the target population, the time origin, the relevant intervention strategies, how intercurrent states are handled, and whether the effect is marginal over post‐randomization histories or conditional on selected histories. Transition‐specific summaries require special care because conditioning on post‐randomization state occupancy may destroy the baseline exchangeability created by randomization. For example, in an illness‐death model, an apparent treatment effect on death may reflect a direct effect on mortality, an indirect effect through preventing or delaying illness, or a change in the composition of patients who survive long enough to enter the illness state. In reversible processes, additional ambiguity arises because the target effect may depend on whether it is defined for first entry into a state, total time spent in a state, recurrence frequency, or transition intensities between states. We now highlight one further issue.

Consider, as a constructed example, the multistate structure depicted in Figure 1, representing a randomized prevention trial evaluating a treatment intended to reduce the risk of developing disease A. At baseline (State 0), treatment is randomly assigned and all participants are free of both diseases A and B. Subsequently, participants may develop disease A (State 2) or disease B (State 1), or die either disease‐free or following one or both diagnoses. We assume, for simplicity, that the preventive treatment protocol targeting disease A remains unchanged after diagnosis of disease B. Such a framework reflects realistic clinical‐trial settings in which multiple, temporally ordered events may occur during follow‐up and competing modes of failure coexist.

Assume that the scientific aim is to estimate the effect of the preventive treatment for disease A on the time to diagnosis of disease A. However, because the hazard functions associated with each transition (i.e., each arrow in Figure 1) are not necessarily identical, the intercurrent events of disease B diagnosis (State 1) and death before disease A diagnosis (States 3 and $3^{\prime \prime }$) must be incorporated into the analysis. Ignoring these processes may lead to bias or to treatment effects that capture various pathways, since transitions between states are dynamically dependent. Such data often tempt investigators to analyze each transition separately. For instance, comparing the State1→State2 transition between treated and untreated participants. Yet, among individuals who have entered State 1, randomization no longer guarantees exchangeability because treatment may influence the probability of reaching that state. In other words, conditioning on posttreatment states violates the assumption that treatment assignment remains independent of future potential outcomes given the observed history. In this case, the counting‐process perspective of Figure 1B of Beyersmann et al. (2025) cannot be applied directly, since the treated and untreated subgroups in State 1 are no longer comparable. Note that the problem is not only with the hazard itself, but with the decision to contrast functionals of the hazard function conditionally on post‐randomization state occupancy. For example, consider the transition probabilities from State 1 to State 2 among those who are in State 1 at 6 months after randomization. Neither the pointwise hazard contrast (for the State1→State2 transition) between treatment arms nor the transition probabilities are likely to contrast balanced and exchangeable treatment groups. A more direct causal approach would define the treatment effect from the baseline‐randomized population, for example, as the effect on the cumulative incidence of disease A by a fixed time, with disease B and death handled as intercurrent events according to the scientific question. Other examples for estimands that can be compared between treatment arms are time spent in state and state‐occupation probabilities at a given time. If the post‐disease‐B transition is itself the target, a well‐defined causal question comparing potential interventions among the same population should be formulated. The original randomization alone might not suffice for identification of such estimands, and additional identification assumptions might be needed and should be judged in the context of the study.

In summary, the growing use of multistate methods in clinical‐trial analysis magnifies both the analytical power and the causal fragility of hazard‐based inference. In clinical‐trial multistate analyses, each transition‐specific hazard summarizes the instantaneous evolution of that transition conditional on the accumulated clinical history, not a universal causal effect that can be transported across states or populations. Extending the discussion of Beyersmann et al. (2025) to this setting emphasizes that, while hazards remain the key identifiable quantities, their causal interpretation is inherently context‐specific and depends on the chosen functional.

## 5 Concluding Remarks

Beyersmann et al. (2025) provide an elegant reconciliation between the identifiability of hazards and their causal interpretation. Our discussion underscores that in many settings, hazards, while indispensable for modeling event‐time data, are inherently context‐dependent. Their interpretation and generalizability depend on the interplay between treatment, censoring, and intercurrent‐event structures.

In clinical‐trial analyses, these considerations highlight the need to move beyond a uniform reliance on hazard ratios and toward estimand‐oriented frameworks that explicitly define the causal question of interest. In competing‐risk settings, this requires stating whether the target is, for example, the total effect on the cumulative incidence of the event of interest, a controlled direct effect under hypothetical elimination of a competing event, a principal stratum effect among those who would not experience the competing event under both treatment values, or a separable effect based on a scientifically plausible decomposition of treatment components.

## Conflicts of Interest

The authors declare no conflicts of interest.

## Data Availability

Data sharing not applicable to this article as no datasets were generated or analyzed during the current study.
